# Case Report: Unveiling the hidden: a rare case of endometrial tuberculosis presenting as peritoneal carcinomatosis

**DOI:** 10.3389/fonc.2025.1607025

**Published:** 2025-07-29

**Authors:** Mariem Garci, Mehdi Makni, Ghada Abdelmoula, Amani Abdeljabbar, Wafa Babay, Nahla Ben Saada, Fatma Dhieb, Linda Hadj Kacem, Nabil Mathlouthi, Cyrine Belghith, Olfa Slimani

**Affiliations:** ^1^ Faculty of Medicine of Tunis, University of Tunis El Manar, Tunis, Tunisia; ^2^ Department A of Gynecology and Obstetrics, Charles Nicolle University Hospital of Tunis, Tunis, Tunisia; ^3^ Faculty of Medicine of Sousse, University of Sousse, Sousse, Tunisia; ^4^ Laboratory of microorganisms and actives biomolecules, Faculty of sciences, University of Tunis El Manar, Tunis, Tunisia; ^5^ Department of Radiology, Charles Nicolle University hospital, Tunis, Tunisia; ^6^ Department of Anatomic Pathology, Charles Nicolle Hospital, Tunis, Tunisia

**Keywords:** endometrial tuberculosis, peritoneal tuberculosis, extrapulmonary tuberculosis, perimenopause, case report

## Abstract

**Background:**

Endometrial tuberculosis (TB) is a rare form of extrapulmonary TB, particularly uncommon in postmenopausal women. Its atypical presentation, characterized by nonspecific symptoms, often leads to misdiagnosis, particularly when it is confused with malignancies. Moreover, peritoneal tuberculosis, although rare, can further complicate the diagnostic process due to its clinical manifestations that resemble those of various cancerous conditions. The coexistence of both endometrial and peritoneal TB in the same patient is particularly unusual and presents a significant diagnostic challenge.

**Case presentation:**

We report the case of a 49-year-old perimenopausal woman who presented with chronic pelvic pain, ascites, and postmenopausal bleeding. Initial imaging raised suspicion for peritoneal carcinomatosis. However, histopathological and microbiological investigations confirmed the diagnosis of endometrial and peritoneal tuberculosis. The diagnosis was established by the detection of acid-fast bacilli and granulomas in the biopsies from the endometrium and peritoneum. The patient was successfully treated with a standard anti-TB regimen, showing a favorable clinical response and gradual resolution of symptoms.

**Conclusion:**

This case underscores the importance of considering tuberculosis in the differential diagnosis of pelvic pathologies, particularly in endemic regions where TB is prevalent. It highlights the need for thorough investigation in cases of atypical pelvic symptoms in patients with risk factors, even in the absence of clear pulmonary symptoms. Including tuberculosis in the differential diagnosis could prevent misdiagnosis and allow for more prompt and appropriate management.

## Introduction

Genital tuberculosis (GTB) accounts for approximately 9–44% of extrapulmonary tuberculosis in women and is diagnosed in 1–19% of women with infertility, depending on geographic region and population studied (1, 2). However, its true prevalence remains underestimated due to its frequently asymptomatic nature.

Its clinical presentation is often nonspecific, ranging from chronic pelvic pain to abnormal uterine bleeding, and may mimic gynecologic malignancies, leading to diagnostic delays (1). This case is unique because it highlights a rare coexistence of endometrial and peritoneal tuberculosis in a 49-year-old woman, initially misdiagnosed as peritoneal carcinomatosis based on imaging findings.

The overlapping clinical and radiologic features with advanced ovarian cancer underscore the importance of including tuberculosis in the differential diagnosis, especially in endemic regions (3). Accurate diagnosis in our case was achieved only through histopathological examination and molecular testing, emphasizing the crucial role of tissue biopsy in distinguishing tuberculosis from malignancy (3).

## Patient information

A 49-year-old perimenopausal woman presented with a 6-month history of chronic pelvic pain, abdominal bloating, unintentional weight loss, and postmenopausal vaginal bleeding. She had no personal or familial history of malignancy or tuberculosis, and no known contact with TB-infected individuals.

## Clinical findings

On examination, mild abdominal distension, lower abdominal tenderness, and moderate ascites were noted. No pelvic masses were palpable. Gynecological examination revealed no cervical abnormalities, and the uterus was of normal size on bimanual exam.

### Timeline

- 6 months prior to presentation: Onset of pelvic pain, abdominal bloating, and weight loss.
- First consultation: Identification of postmenopausal bleeding, elevated CA-125, and imaging findings suggestive of peritoneal carcinomatosis.
- Initial diagnostic approach: Transvaginal ultrasound followed by attempted outpatient endometrial sampling with Pipelle (non-diagnostic due to scant tissue).
- Subsequent steps: Decision for diagnostic laparoscopy due to diagnostic uncertainty and concern for advanced malignancy.
- Histopathologic confirmation: Endometrial and peritoneal biopsies consistent with tuberculosis.
- Treatment initiation: Anti-tuberculous regimen started.
- 3 months post-treatment: Resolution of bleeding and ascites.
- 12-month follow-up: Asymptomatic status, normalized CA-125, and regression of lesions on imaging.

### Diagnostic assessment

Initial laboratory evaluation revealed an elevated CA-125 level of 265 U/mL, while CEA and CA 19–9 were within normal limits, and HIV serology was negative. Transvaginal ultrasonography was performed; however, images could not be archived at the time of the examination. The ultrasound demonstrated irregular endometrial thickening with hyperechoic foci. Considering the patient’s age and clinical presentation, an endometrial biopsy using a Pipelle device was attempted but yielded insufficient tissue for diagnosis. Due to persistent abnormal bleeding and imaging findings suggestive of peritoneal carcinomatosis, further diagnostic evaluation was deemed necessary.

**Figure 1:**
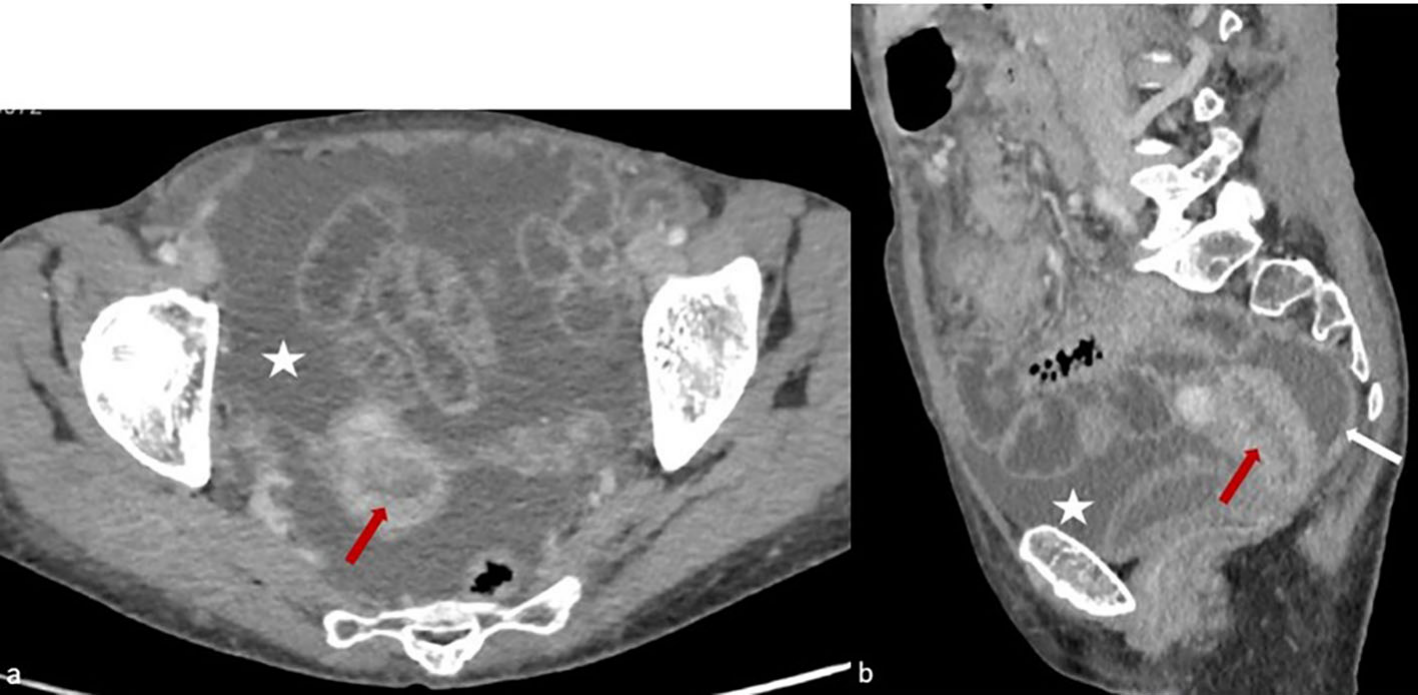
Axial **(a)** and sagittal **(b)** sections of a contrast-enhanced abdominopelvic CT scan obtained during the portal venous phase, demonstrating hypodense and irregular endometrial thickening (red arrow), accompanied by a significant peritoneal effusion (white star) and thickening of the pelvic peritoneal layers (white arrow).

**Figure 2:**
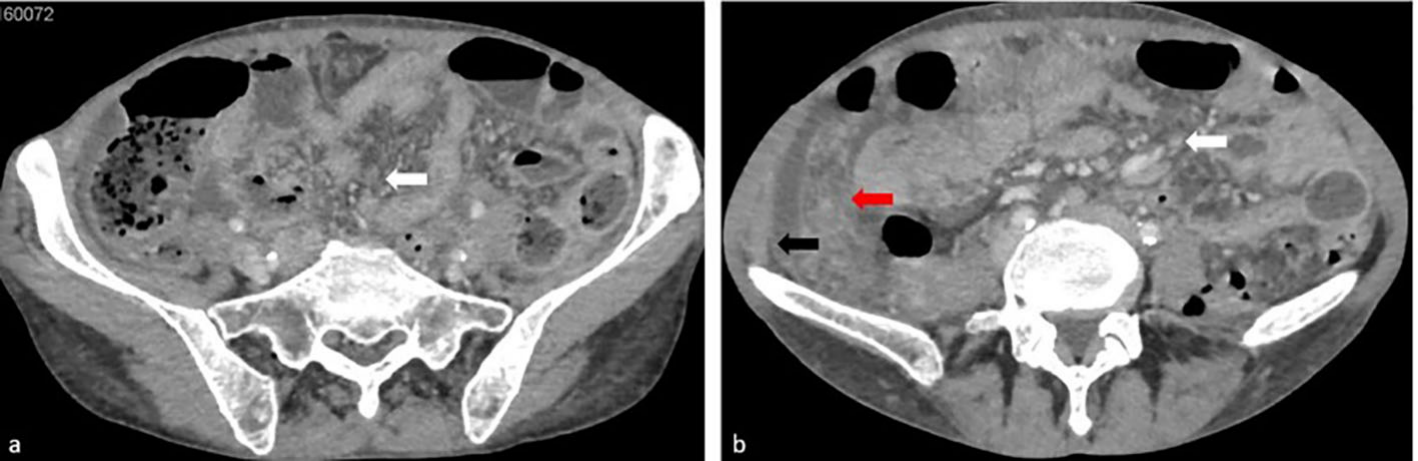
Axial contrast-enhanced CT images acquired during the portal venous phase reveal micronodular infiltration of the peritoneal fat, characteristic of an ‘omental cake’ morphology (red arrow), alongside peritoneal soft tissue nodules (white arrow) with peritoneal effusion and diffuse thickening of the peritoneal layers (black arrow).

Contrast-enhanced CT revealed ascites, omental caking, peritoneal thickening, and nodularity (**Figures 1**, **2**). Although malignancy was suspected, peritoneal fluid cytology showed no malignant cells, and tumor markers were not strongly suggestive. A formal staging workup, including thoracic imaging, was performed. Multidisciplinary tumor board discussion favored a diagnostic laparoscopy in the absence of a conclusive diagnosis by noninvasive means.

**Figure 3:**
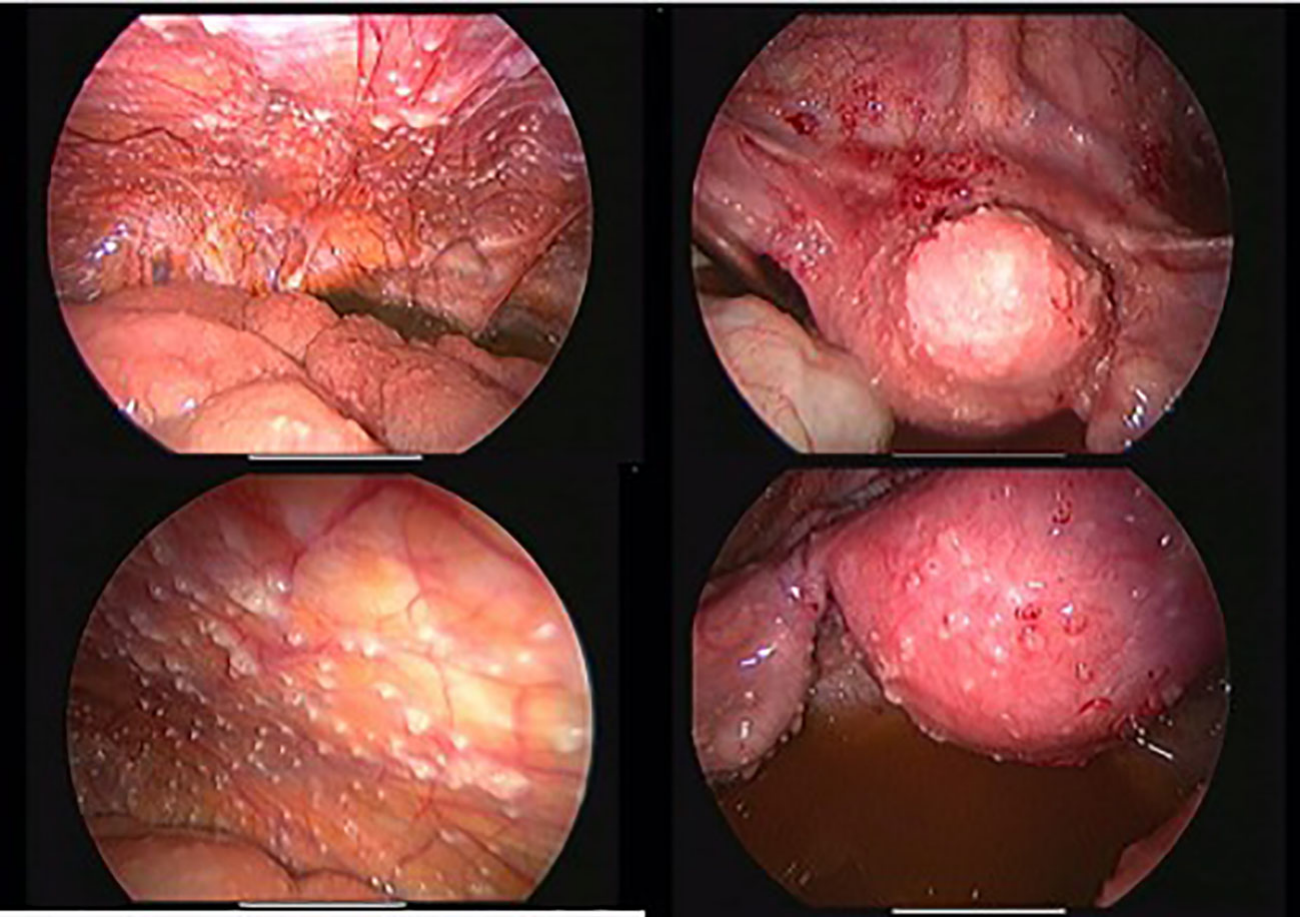
Laparoscopic findings: white peritoneal tubercles, adhesions, and granulomas confirming the diagnosis.

**Figure 4:**
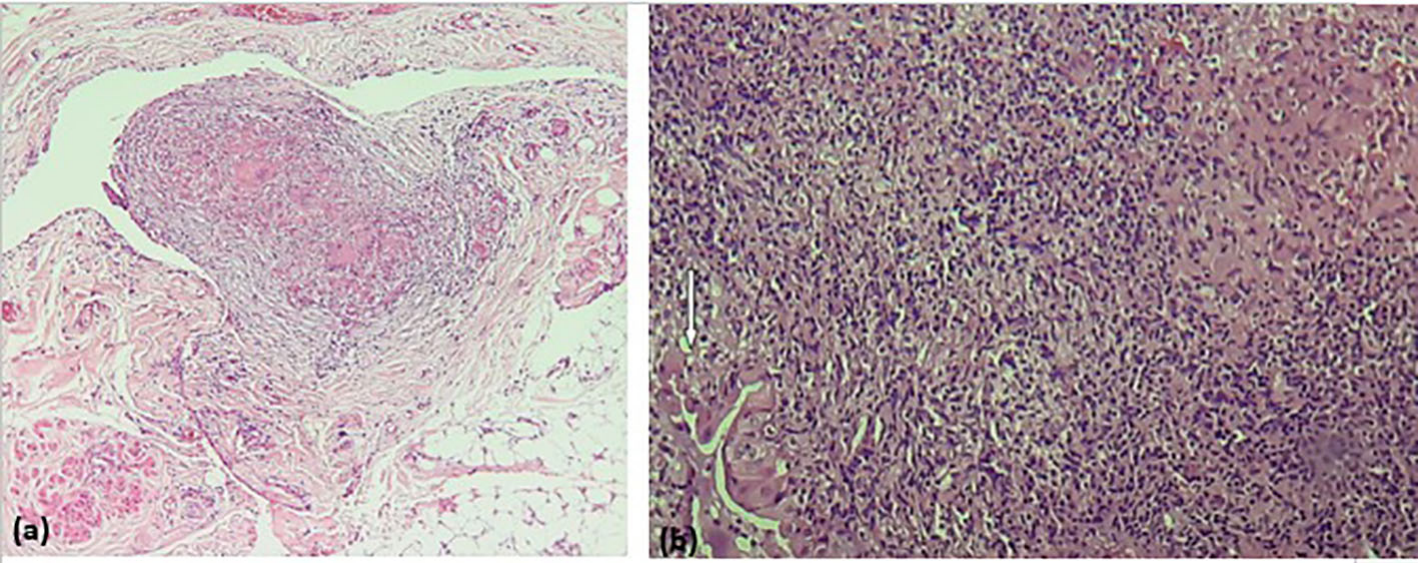
**(a)** Peritoneal tissue with epithelioid and gigantocellular granulomas; **(b)** endometrial mucosa largely rearranged by a dense inflammatory and granulomatous infiltrate of epithelioid and gigantocellular granulomas associated with numerous neutrophils and eosinophils. The few residual endometrial glands (White arrow) are the site of exocytosis of inflammatory elements, without hyperplasia.

Diagnostic laparoscopy (**Figure 3**) revealed classic signs of peritoneal tuberculosis: numerous whitish tubercles, dense adhesions, and granulomatous plaques. Biopsies were taken from the peritoneum and endometrium. Histopathological analysis confirmed caseating granulomas with Langhans giant cells, characteristic of tuberculosis (**Figure 4**). GeneXpert MTB/RIF assay on peritoneal fluid was positive for *Mycobacterium tuberculosis*, and cultures were initiated. No evidence of malignancy or dysplasia was identified on histology, and thorough sectioning ruled out synchronous endometrial carcinoma with granulomatous reaction.

### Diagnosis

Endometrial tuberculosis with associated peritoneal tuberculosis, mimicking peritoneal carcinomatosis.

### Therapeutic intervention

The patient was treated with standard first-line anti-tuberculous therapy:

- Rifampicin 600 mg/day
- Isoniazid 300 mg/day
- Pyrazinamide 1500 mg/day
- Ethambutol 1200 mg/day

This intensive phase lasted for two months, followed by a four-month continuation phase with rifampicin and isoniazid. No significant adverse events were reported during treatment, and adherence was confirmed through monthly follow-ups.

### Follow-up and outcomes

Three months after initiating treatment, postmenopausal bleeding had ceased, and the patient reported marked symptomatic improvement. Repeat CA-125 level had decreased to 28 U/mL. Given the favorable clinical and biological response, no follow-up imaging was performed. At one-year follow-up, the patient remained asymptomatic, with normal laboratory parameters and a normal gynecologic examination.

### Patient perspective

The patient expressed optimism following her diagnosis and treatment. She reported significant improvement in symptoms, including reduced pelvic pain and postmenopausal bleeding, which had previously affected her quality of life. Satisfied with the care provided, she remains hopeful for a full recovery, feeling reassured about her health moving forward.

### Informed consent

The patient provided written informed consent for publication of her clinical details and images.

## Discussion

Endometrial tuberculosis (TB) is an uncommon manifestation of genital TB, often presenting with nonspecific symptoms such as abnormal uterine bleeding, pelvic pain, or infertility. Its rarity and variable clinical presentation pose a diagnostic challenge, especially when it mimics malignancies such as peritoneal carcinomatosis. In this case, the patient exhibited extensive peritoneal involvement, ascites, and imaging features highly suggestive of metastatic ovarian or endometrial cancer, leading to an initial misdiagnosis.

The differentiation between endometrial TB and peritoneal carcinomatosis is critical, as their management and prognosis differ significantly. Imaging modalities such as ultrasound, CT, and MRI may reveal ascites, omental caking, and peritoneal thickening—findings commonly associated with peritoneal carcinomatosis (1, 2). However, these radiological features are not pathognomonic and can also be seen in infectious diseases, including TB peritonitis (3). In our case, despite the strong suspicion of malignancy, histopathological examination and microbiological studies confirmed TB, emphasizing the importance of tissue diagnosis in ambiguous presentations.

Several recent reports have highlighted a global increase in extrapulmonary tuberculosis (EPTB), particularly in immunocompromised individuals—including those with HIV, diabetes, or receiving immunosuppressive therapy—as well as in populations residing in TB-endemic regions. According to the WHO Global Tuberculosis Report 2022, extrapulmonary forms now represent up to 20–25% of all TB cases worldwide, and over 50% in some high-burden regions, such as sub-Saharan Africa and Southeast Asia (4). The rising prevalence is attributed not only to increased HIV co-infection but also to improved diagnostic awareness and use of molecular testing methods like GeneXpert (5).

The hematogenous or lymphatic spread of Mycobacterium tuberculosis to the endometrium and peritoneum can occur either as a primary genital infection or secondary to latent TB reactivation (6). In our patient, the absence of pulmonary involvement and the lack of typical systemic symptoms, such as fever or weight loss, further complicated the diagnosis.

From a clinical reasoning standpoint, this case illustrates a typical example of anchoring bias: the diagnostic process was initially oriented toward gynecologic malignancy due to the patient’s perimenopausal status, ascites, and imaging suggestive of carcinomatosis. The absence of endometrial sampling prior to laparoscopy—such as a simple Pipelle biopsy—represented a missed opportunity for a less invasive diagnosis. Moreover, the absence of elevated CA-125 or constitutional symptoms did not prompt a reconsideration of alternative diagnoses such as TB. This highlights the importance of maintaining a broad differential, especially in endemic settings, and guarding against premature closure.

The decision to proceed with diagnostic laparoscopy was made in the context of extensive peritoneal involvement with inconclusive noninvasive findings. In our institution, laparoscopy is preferred when peritoneal carcinomatosis is suspected but cannot be confirmed radiologically or cytologically. It provides direct visual inspection and biopsy, enabling both diagnostic accuracy and avoidance of unnecessary extensive surgery.

Endometrial tuberculosis can closely mimic advanced gynecologic malignancies due to its tendency to cause adhesions, ascites, and mass-like lesions (7). Previous studies have reported cases where endometrial or peritoneal tuberculosis presented similarly to ovarian cancer, often leading to unnecessary surgical interventions (8, 9). Therefore, a high index of suspicion is essential, especially in patients from endemic regions or those with risk factors such as prior TB exposure or immunosuppression (10). In a retrospective cohort study of 138 patients initially suspected of ovarian cancer, 5.7% were ultimately diagnosed with pelvic tuberculosis (11). These patients exhibited clinical symptoms and radiological features suggestive of malignancy, including complex adnexal masses, ascites, and elevated CA-125 levels, which complicated the differential diagnosis.

This diagnostic challenge is particularly concerning because genital tuberculosis may lead to unnecessary and sometimes extensive surgical procedures, such as exploratory laparotomies or adnexectomies, driven by the initial suspicion of cancer. However, appropriate anti-tuberculous treatment usually results in complete resolution without the need for invasive surgery (11).

These findings emphasize the importance of comprehensive diagnostic assessment and maintaining vigilance for tuberculosis, particularly in areas with high prevalence. Use of additional diagnostic modalities, such as endometrial or laparoscopic biopsy for histological confirmation, along with specific imaging techniques, should be prioritized to avoid overtreatment (11).

The cornerstone of treatment for genital tuberculosis remains a prolonged course of anti-tuberculous therapy (ATT), typically comprising a multidrug regimen of rifampicin, isoniazid, pyrazinamide, and ethambutol for an initial intensive phase, followed by a continuation phase with rifampicin and isoniazid (12). According to WHO guidelines, the standard duration for drug-susceptible extrapulmonary TB, including genital and peritoneal forms, is six months, although longer regimens may be considered in complex or relapsing cases (4, 12).

Several studies have demonstrated that ATT alone is highly effective in achieving microbiological cure and symptom resolution, even in advanced presentations involving ascites, pelvic adhesions, or mass-like lesions that mimic malignancy (13). A prospective cohort study by Sharma et al. (13) involving 112 women with genital TB in endemic regions found a complete clinical response in over 90% of cases after six months of ATT, with resolution of bleeding, pain, and radiologic abnormalities.

In our patient, first-line anti-tuberculous therapy resulted in marked clinical improvement within three months, supporting both the diagnosis and the efficacy of medical management. Importantly, early diagnosis and timely initiation of therapy can prevent irreversible tissue damage, preserve reproductive function in younger women, and avoid unnecessary surgical interventions, especially when the disease mimics gynecologic malignancies.

## Conclusion

This case highlights the diagnostic challenge of endometrial and peritoneal TB in perimenopausal women, where symptoms often mimic malignancy. A high index of suspicion, thorough histopathological evaluation, and early initiation of treatment are essential for optimal outcomes. Multidisciplinary collaboration between gynecologists, radiologists, and infectious disease specialists is crucial to prevent misdiagnosis and unnecessary surgical interventions.

## Data Availability

The original contributions presented in the study are included in the article/supplementary material. Further inquiries can be directed to the corresponding author/s.
